# Research—A Challenge to the Press

**Published:** 1893-02-18

**Authors:** 


### The Hospital

AN INSTITUTIONAL JOURNAL OF

**Science, Medicine, Nursing, and Philanthropy.**

*For Hospitals, Asylums, Infirmaries, Dispensaries, Medical Practitioners, Students, Nurses, and the Charitable Public.*

VOL. XIII.—No. 334.] [FEB. 18, 1893.

### RESEARCH—A CHALLENGE TO THE PRESS.

MR. LAWSON TAIT, at the meeting held last week at Birmingham on behalf of the proposed British Institute of Preventive Medicine, expressed his entire approval of the "objects of the Institute." It is difficult to see how any humane man, surgeon or otherwise, could do other than approve of those objects. For what are the objects but efforts to do everything which lies within the power of science to prevent disease, and, where it cannot be prevented, to cure it? Of course there is a sense in which it might be supposed that medical men would not naturally wish to prevent disease. Diseases of all kinds are obviously "grist to the medical mill," and the ordinary maxims of business, or even of political economy, do not contemplate the possibility of the existence of a class of men who deliberately, and of set purpose, lay their plans to "ruin their own markets." But this is what medical men habitually do, and what they receive little credit for doing. It is also what the British Institute of Preventive Medicine has for its chief object, in spite of which, however, the promoters of the Institute are covered with opprobrium and abuse by a class of people who ought to be the very last to say a single opposing word on the subject.

The physicians and medical scientists of the present day are under the necessity of apologising for striving to render to the community and all the individuals composing it, important services. We do not complain of this; public benefactors of almost all classes have had to pass through preliminary periods of opprobrium before they and their works have been publicly acknowledged. Doctors are not the persons to whine because ignorance assails them ignorantly. But it is desirable, in the interests of the public itself, that doctors shall take every opportunity of appealing to reason and of removing dangerous prejudice. Such an appeal to reason we now propose to make, but on somewhat new lines.

Research, physiological research, it is manifest, is being conducted on an extensive scale, on the European continent and in America; and there is no doubt whatever that it will continue to be so conducted. It is desirable, in the interests alike of researchers, of experimental animals, and of good morals, that such research shall be of the most practically important character, and that it shall not be extended beyond such limits as the necessities of the case demand. Now the most practical, the most sober, and the most humane of all researchers are undoubtedly the scientists of these islands. The reason for this we need not now stay to inquire. Probably it is that the condition of public feeling is more sensitive and sympathetic generally in this country than elsewhere, and that our physiological researchers reflect the general tone of their fellow-countrymen. Be that as it may, there is no doubt of the fact that British researchers show a scrupulousness and a careful kindness towards experimental animals which are by no means universal among physiologists.

Upon these acknowledged facts we found the contention that the animal world would be more kindly and humanely treated as a whole if British researchers were endowed with such freedom and resources as would enable them to take a leading place in physiological research, and so to control, by example and proved results, the exuberant extravagance of young and less scrupulous foreigners. It is plain that physiological research is not a parochial question, and that it should not be considered parochially. We must consider the subject from the standpoint of the whole world's experimental animals, and the whole world's physiologists. If by giving freedom and facilities to British researchers we can seriously diminish the numbers of the animals experimented upon throughout the world, and mitigate the painfulness of experimental operations generally, would it not be serving the very objects which the anti-vivisectors themselves have in view if we were forthwith to give to our own researchers that freedom and those facilities?

It is not e[illegible] to understand the attitude of the British publi[illegible]owards physiological researchers. Everybody is eager to have the newest medical science at his own bedside when he is sick. If any physician or surgeon were to proclaim to the world that he proposed to conduct his practice according to the principles and methods of the first century, he would speedily find his consulting-room as lonely as an African desert. But how is science to be advanced except by the methods proper to science? And of all the methods which science employs, is not experiment the most conclusive, is it not the most absolute *sine quâ non* of demonstration? One cannot help wondering what was the condition of mind of Mr. Lawson Tait when he expressed approval of the "objects" of the British Institute! If he approve the objects, can he avoid also approving the methods? Apart from its methods the British Institute has no objects. It does not profess to be content with the empirical methods of the bedside. These, we admit, are invaluable, and, indeed, indispensable; but they are not the methods of definite and demonstrable science. It seems to us to be in the highest degree illogical and inconsequent for the British people to demand all the newest results of the newest science, but to decline to let our scientists pay any part of the price of such results. Nay, it is not merely illogical, it is mean, if it be not indeed hypocritical. If we will not have anything to do with researchers and research, then let us have the courage of our scruples, and decline to have anything to do with the fruits of their labours.

The leaders of public opinion have not, in our judgment, done justice to medical scientists in this matter. They have been like the Laodiceans, "neither cold nor hot." It is impossible to doubt that their minds have been with the researchers. But why then have their tongues been silent and their pens still?

We challenge the general press to a controversy on this subject. Do newspaper writers believe in scientific medicine; or do they prefer the methods of the empiric and the quack? If they elect to stand by science and experiment, why do they not take the trouble to go to head-quarters and thoroughly inform themselves upon the merits of the British Institute question? Are we to believe that the general press sets absolutely no value upon the labours and results of Pasteur and of Virchow, of Lister and of Koch? It is to pay the press a sorry compliment to suppose either that it does not understand the significance of modern physiological discoveries, or that it is indifferent to them. But are we not almost compelled to adopt either the one alternative or the other? At any rate, we see the proposal for a British Institute of Preventive Medicine hung up like a defunct Parliamentary Bill, that nobody cares either to see or to hear of any more. The result of this appears to be that medical men will not only be left to strive to ruin their own labour markets by the prevention of disease; but they will have to put their hands into their pockets to pay for the prevention as well as to lose the profits of the cure. This may be "good business" from the general public's point of view, but it cannot be said to be generous or in any way worthy of the character of John Bull. Our challenge to the press is that it shall abandon the Laodicean attitude and do either the one thing or the other; either take up the anti-vivisector's position, and denounce experimental research through all its moods and tenses, or join the pioneer band of progressive science, and help to place England in her rightful position in the forefront of every movement that is scientific, enlightened, and humane.

